# Supplementary material for: Composition and stage dynamics of mitochondrial complexes in Plasmodium falciparum
Source: Nat Commun. 2021 Jun 21;12:3820. doi: 10.1038/s41467-021-23919-x (PMC8217502; doi:10.1038/s41467-021-23919-x)
Supplement: Supplementary file 1 — Supplementary Information [file 41467_2021_23919_MOESM1_ESM.pdf]

Supplementary Information

Composition and stage dynamics of mitochondrial complexes in *Plasmodium falciparum*

Felix Evers[1], Alfredo Cabrera-Orefice[2,3], Dei M. Elurbe[3], Mariska Kea-te Lindert[4,5], Sylwia D. Boltryk[6,7], Till S. Voss[6,7], Martijn A. Huynen[3], Ulrich Brandt[2,8] & Taco W.A. Kooij[1*]

[1]Department of Medical Microbiology, Radboudumc Center for Infectious Diseases, Radboud Institute for Molecular Life Sciences, Radboud University Medical Center, PO Box 9101, 6500 HB Nijmegen, Netherlands. [2]Radboud Institute for Molecular Life Sciences, Radboud University Medical Center, PO Box 9101, 6500 HB Nijmegen, The Netherlands. [3]Centre for Molecular and Biomolecular Informatics, Radboud Institute for Molecular Life Sciences, Radboud University Medical Center, PO Box 9101, 6500 HB Nijmegen, the Netherlands. [4]Electron Microscopy Center, RTC Microscopy, Radboud Institute of Molecular Life Sciences, Radboud University Medical Center, Geert Grooteplein 6525 GA Nijmegen, The Netherlands. [5]Department of Cell Biology, Radboud Institute of Molecular Life Sciences, Radboud University Medical Center, Geert Grooteplein 6525 GA Nijmegen, The Netherlands [6]Department of Medical Parasitology and Infection Biology, Swiss Tropical and Public Health Institute, Basel, Switzerland. [7]University of Basel, Basel, Switzerland. [8]Cologne Excellence Cluster on Cellular Stress Responses in Aging-Associated Diseases (CECAD), University of Cologne, Cologne, Germany. *Correspondence and requests for materials should be addressed to T.W.A.K. (email: taco.kooij@radboudumc.nl).

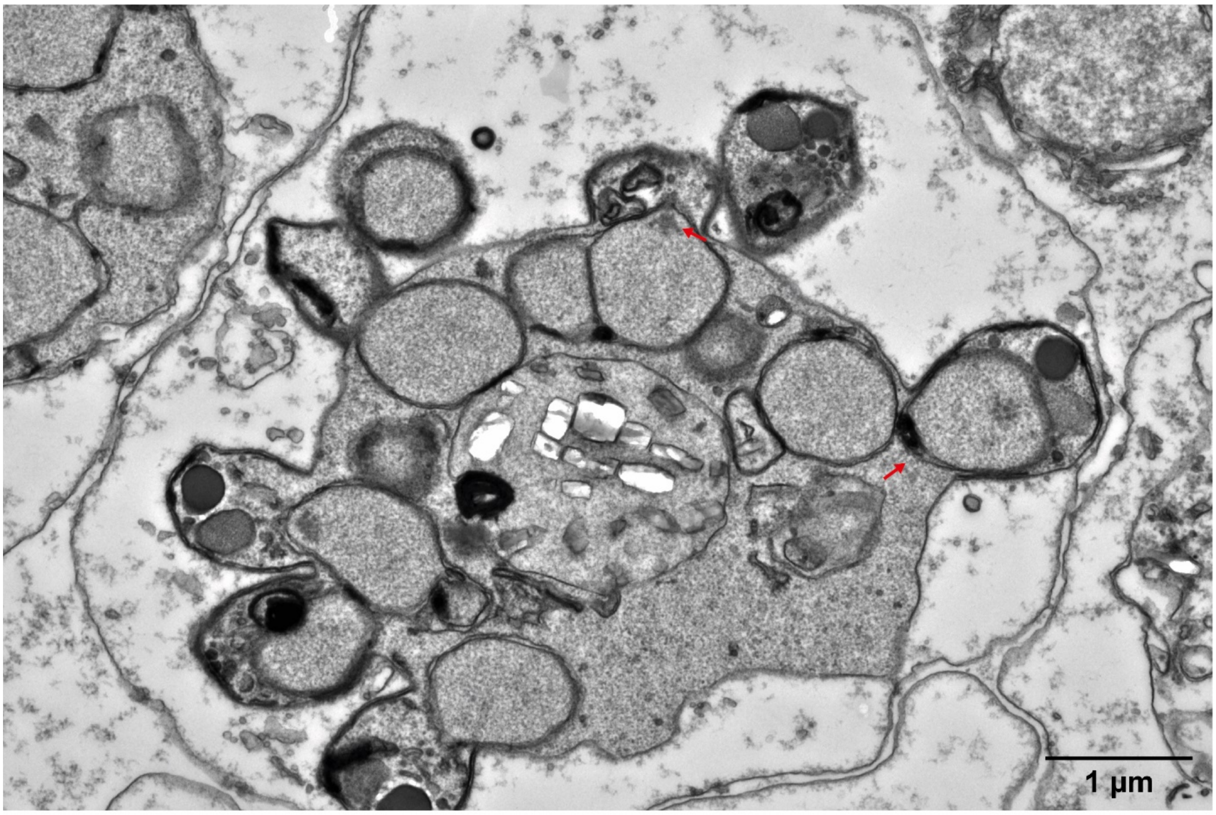

Supplementary Figure 1. Saponin-lysed infected red blood cells containing segmenting schizont. Nuclei appear to enter merozoite compartment after organelles, closing off the compartment after entering (red arrows). More electron-dense part of nucleus during entrance process also potentially indicates directed “pulling” of the nucleus. Two biological samples of ABS lysed with saponin were processed in this way and observations were consistent across both.

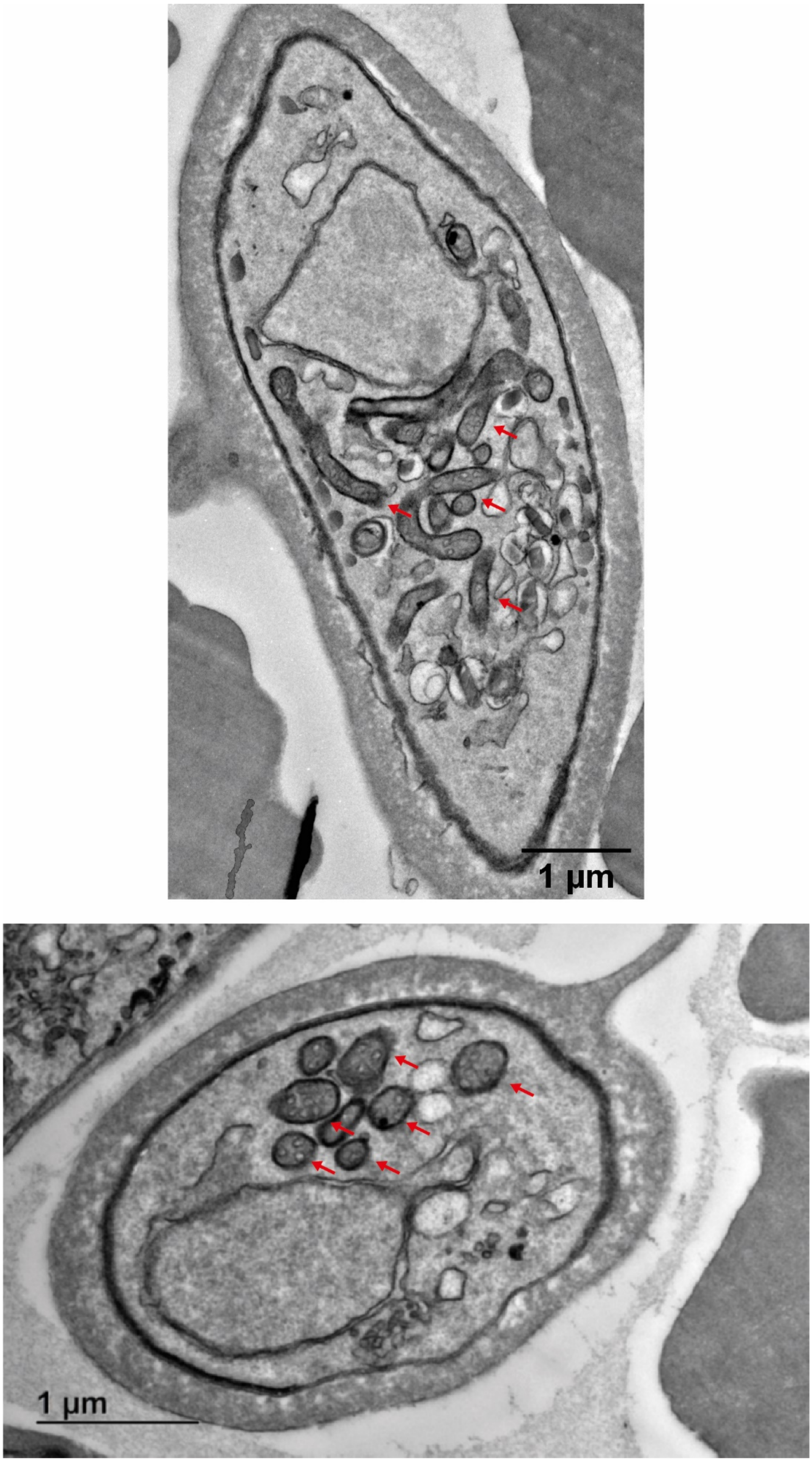

Supplementary Figure 2. Cristate mitochondrial sections cover large proportion of mature gametocytes. Red arrows indicate mitochondrial sections. Upper panel depicts longitudinal section of a gametocyte and bottom panel depicts lateral section of a gametocyte. Three biological replicates of gametocytes were imaged and observations were consistent across all.

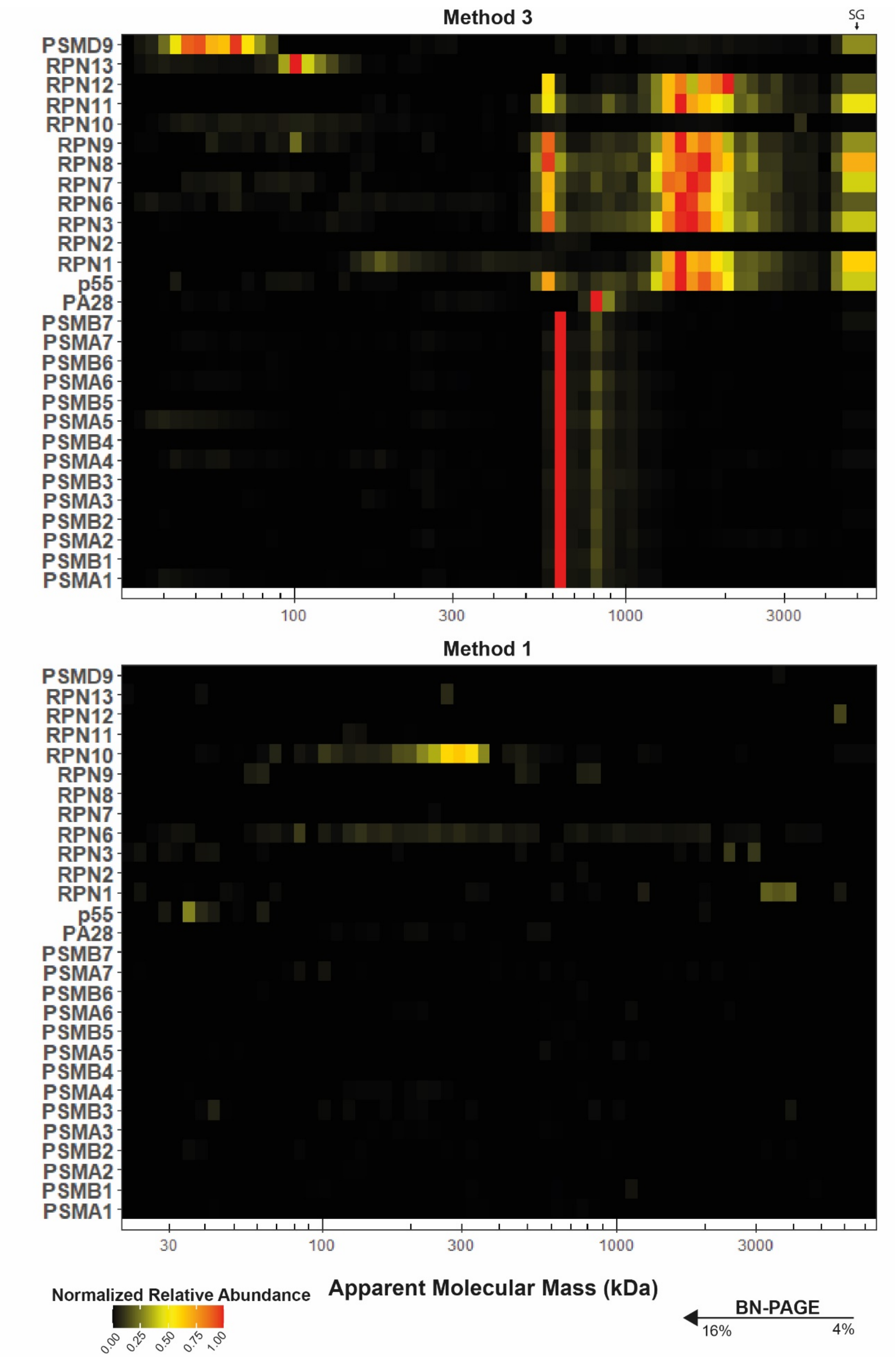

Supplementary Figure 3. Differential presence of assembled proteasome components. Upper panel depicts abundance of proteasome components in sample ABS3D (method 3), lower panel depicts abundance in sample ABS1Da (method 1). Samples were normalized based on highest iBAQ value for each protein group between the two samples. Lack of proteasome detection when homogenate was treated with saponin suggests either reduced cytosolic contaminants or a specific detergent-complex interaction upon saponin treatment.

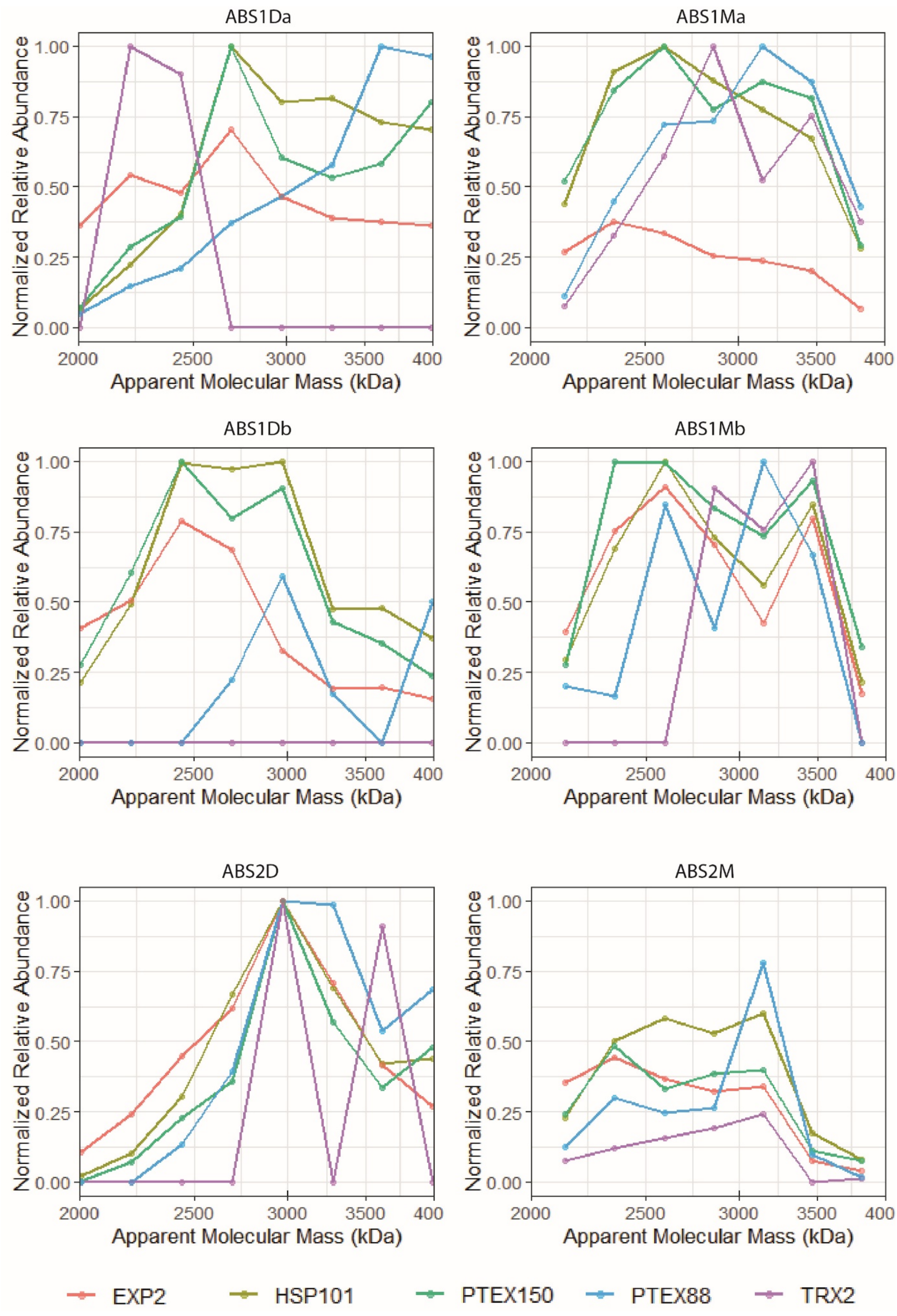

Supplementary Figure 4. Heterogeneous comigration of PTEX88 and TRX2 with PTEX core components. Proteins were normalized based on highest iBAQ value in 1,500-4,000 kDa mass range.

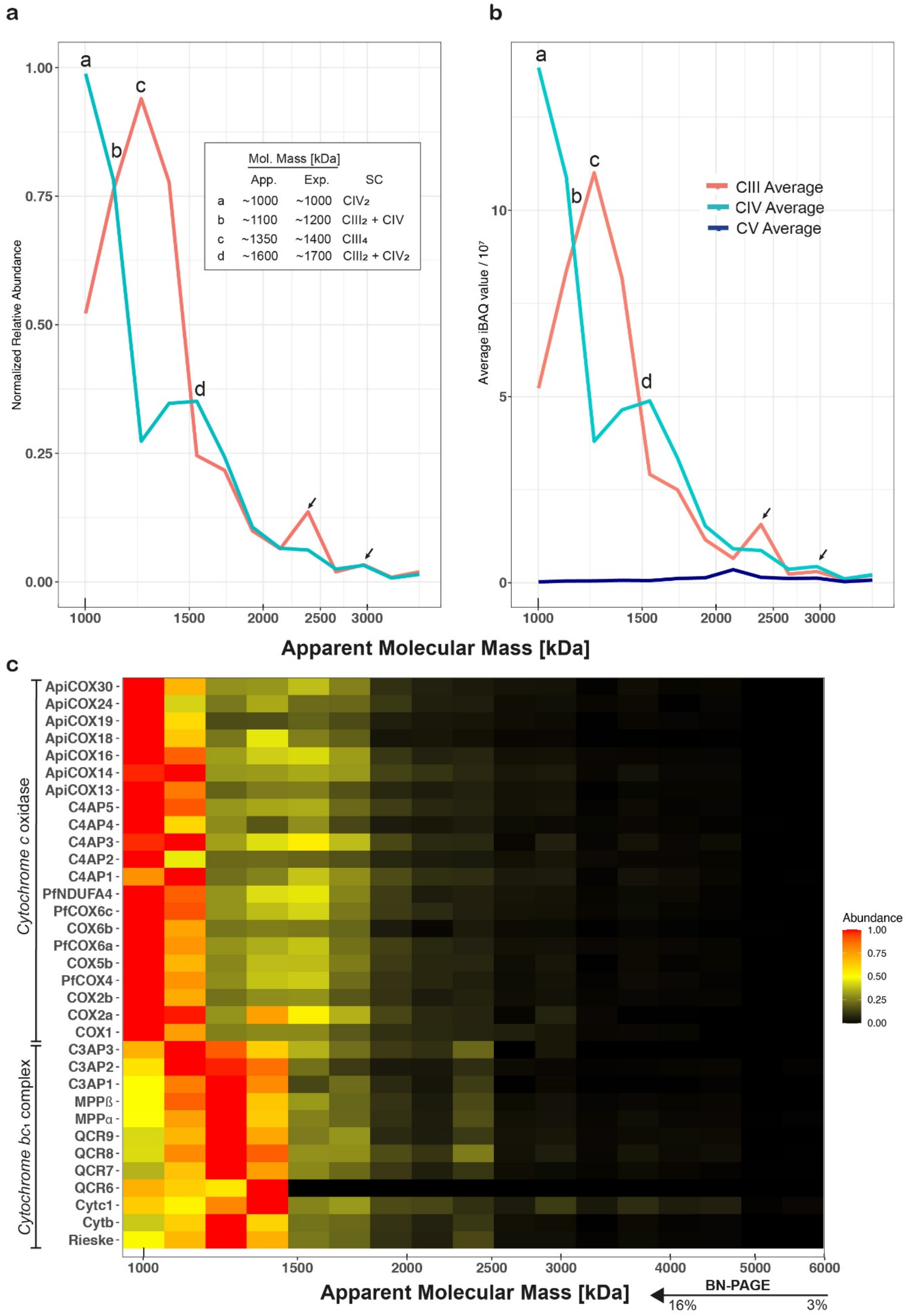

Supplementary Figure 5. 3-16% MDa profiles to investigate putative supercomplexes in stage V gametocytes (**a**) Average normalized abundance of CIII and CIV components in the 1-4 MDa mass range. (**b**) Average iBAQ value / 1000 of CIII, CIV, and CV components in the 1-4 MDa mass range. (**c**) Heatmap of normalized abundance of CII and CIV components in the 1-4 MDa mass range.

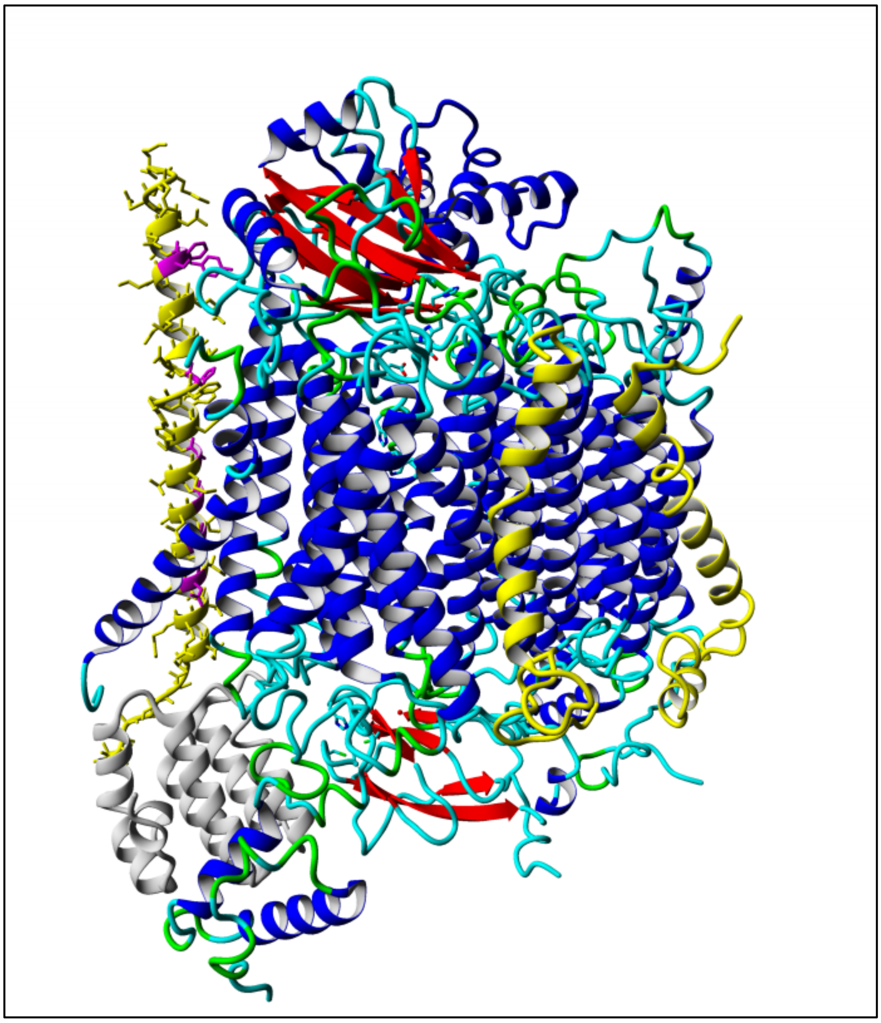

Supplementary Figure 6. CIV from *Saccharomyces cerevisiae*[1]. Subunits that were lost in the evolution to *P. falciparum,* or whose homology to *P. falciparum* proteins is barely detectable, are in yellow. The grey subunit on the bottom-left (named COX6 in *S. cerevisiae* that corresponds to COX5A in Metazoa) is present in *S. cerevisiae* but is an evolutionary addition that is specific to the opisthokonts and was thus never "lost". The yellow subunit on the left (COX9 in *S. cerevisiae* that corresponds to COX6C in Metazoa) is poorly conserved in *P. falciparum*. However, the residues in magenta, which appear to interact with other proteins of the complex, are conserved. The subunits that date back to the last eukaryotic common ancestor and for which no homologues could be detected in *P. falciparum* are COX8 (COX7C in Metazoa) in the middle and COX7 (COX7A in Metazoa) on the right. Visualization done with Yasara[2].

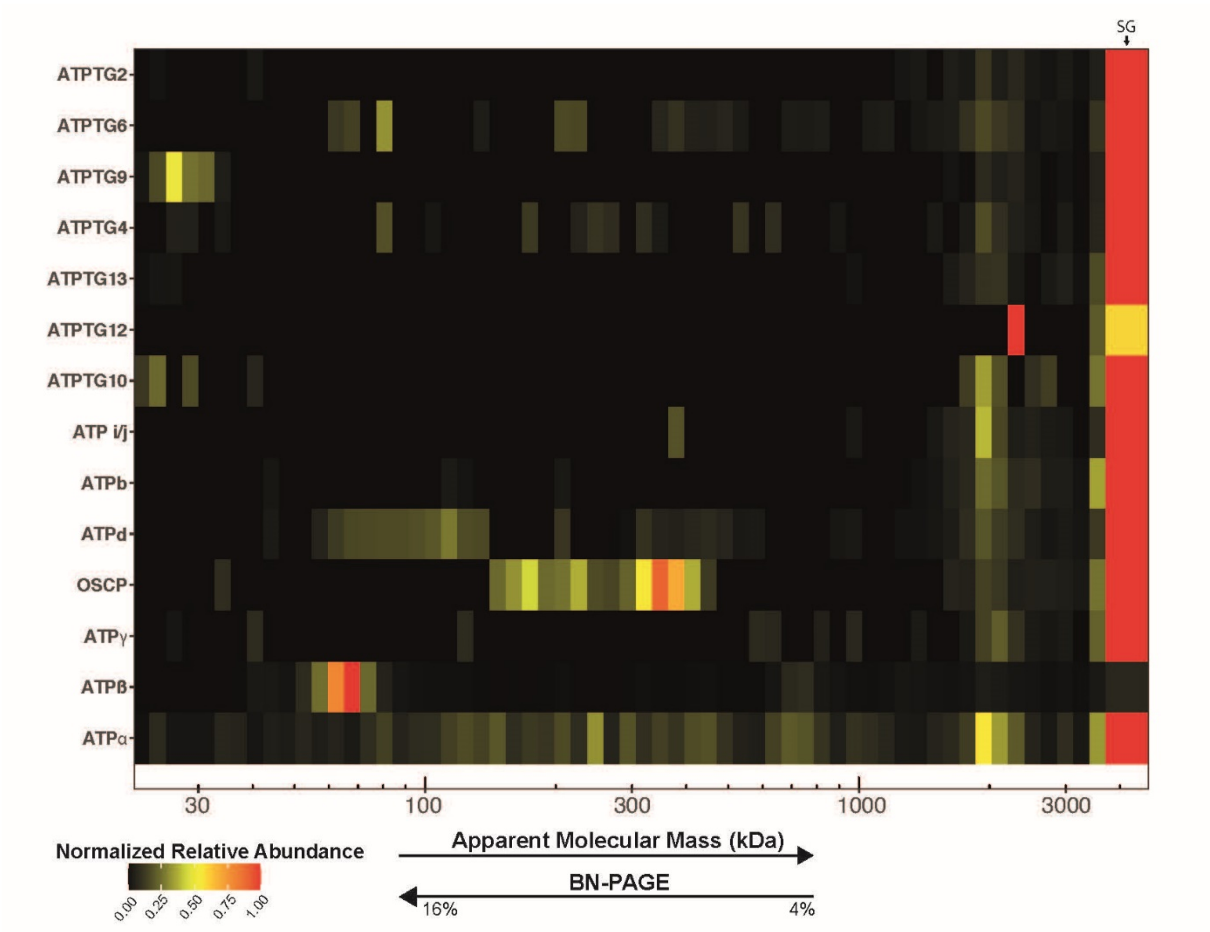

Supplementary Figure 7. Migration pattern of ATP synthase components in ABS3D. Heatmap showing comigration of putative ATP synthase components in sample ABS3D. Black arrow indicates position of stacking gel. Migration pattern matches observation from gametocyte data.

15. NP_566077.1 succinate dehydrogenase subunit 4 [Arabidopsis thaliana]

Probability: 22%, E-value: 100, Score: 22.43, Aligned cols: 55, Identities: 15%, Similarity: 0.085, Template Neff: 3.7

```
Q ss_pred              HHHHHHHHHHHHHHHHHHHHhcCCChHHHHHHHHHHHHhcccccccHHHHHHHHH
Q PF3D7_1448900    19  RSLTMSLYFIGISTVSWFICTTSRRKEWADIMLDYVHHKRCSFLSNSWASFLKRL   73 (75)
Q Consensus       19  ~~l~~s~~~l~~St~~~~~~t~~rrKEWvdIlLDY~hhKR~~~lsn~wd~~~r~l   73 (75)
                       ..+..+..+-.+..+......---.-..-+|+.||+|++-....-...-.++-.+
T Consensus       87  ~~LFss~~~kIltiL~IIsvf~Ha~LGmqvIIEDYVH~~~~RlvlLili~Lf~iv   141 (151)
T NP_566077.1     87  SRQSSSRGYTNGSFLRKIPVVFHIHEGMEEILADYVHQEMTRNLIVMSLGLFQII   141 (151)
T ss_pred              HHHhcCHhHHHHHHHHHHHHHHHHHHHHHHHHHhCChHHHHHHHHHHHHHHHHHH
```

1. NP_566077.1 succinate dehydrogenase subunit 4 [Arabidopsis thaliana]

Probability: 41.47%, E-value: 0.00063, Score: 24.85, Aligned cols: 22, Identities: 27%, Similarity: 0.409, Template Neff: 4.641

```
Q ss_pred                   ChHHHHHHHHHHHHHhcccccc
Q PF3D7_1448900        42   RRKEWADIMLDYVHHKRCSFLS   63 (75)
Q Consensus           42   rrKEWvdIlLDY~hhKR~~~ls   63 (75)
                            -.-...+|+.||+|++-...+.
T Consensus           110  ~~~g~~~il~DYvh~~~~r~~~   131 (151)
T NP_566077.1         110  IHEGMEEILADYVHQEMTRNLI   131 (151)
T ss_pred                   HHHHHHHHHHHhCCcHHHHHHH
```

Supplementary Figure 8. Alignment of PF3D7_1448900 with SDH4 from *Arabidopsis thaliana*. The alignment was obtained by searching with the PF3D7_1448900 sequence against the profiles of *A. thaliana* using HHpred with default settings (Upper panel). If a pairwise search with SDH4 from *A. thaliana* is performed, the e-value improves and the alignment highlights a conserved DY motif. This DY motif has been associated with the binding of quinone in other species.

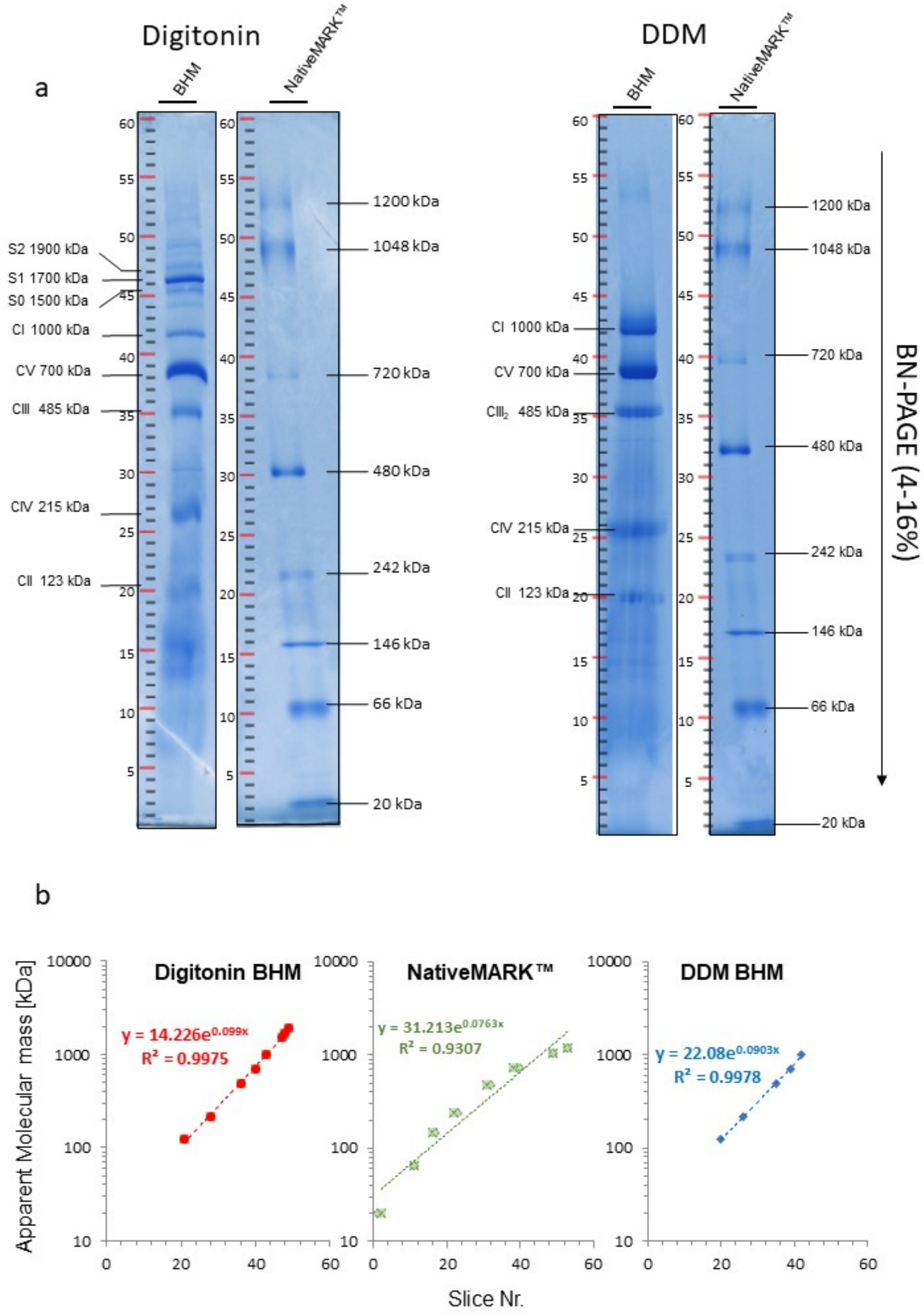

Supplementary Figure 9. Comparing bovine heart mitochondria (BHM) to commercial marker for mass calibration. (**a**) Coomassie stained BHM, solubilized with either DDM or digitonin and NativeMark^{TM} (Invitrogen ; LC0725) separated on 4-16% BN-PAGE. Ruler indicates the slicing of corresponding complexome profiles. Band assignments for BHM are based on prior research[3] and in-house analysis of BHM by complexome profiling. Band assignment for NativeMARK^{TM} is based on manufacturer's documentation. Neighbouring lanes were run on the same gel. The experiment was performed once for each detergent. (**b**) Corresponding mass calibration curves. NativeMARK^{TM} does not follow expected exponential mass/migration relationship at higher apparent masses and generally mass assignments differ greatly from BHM.

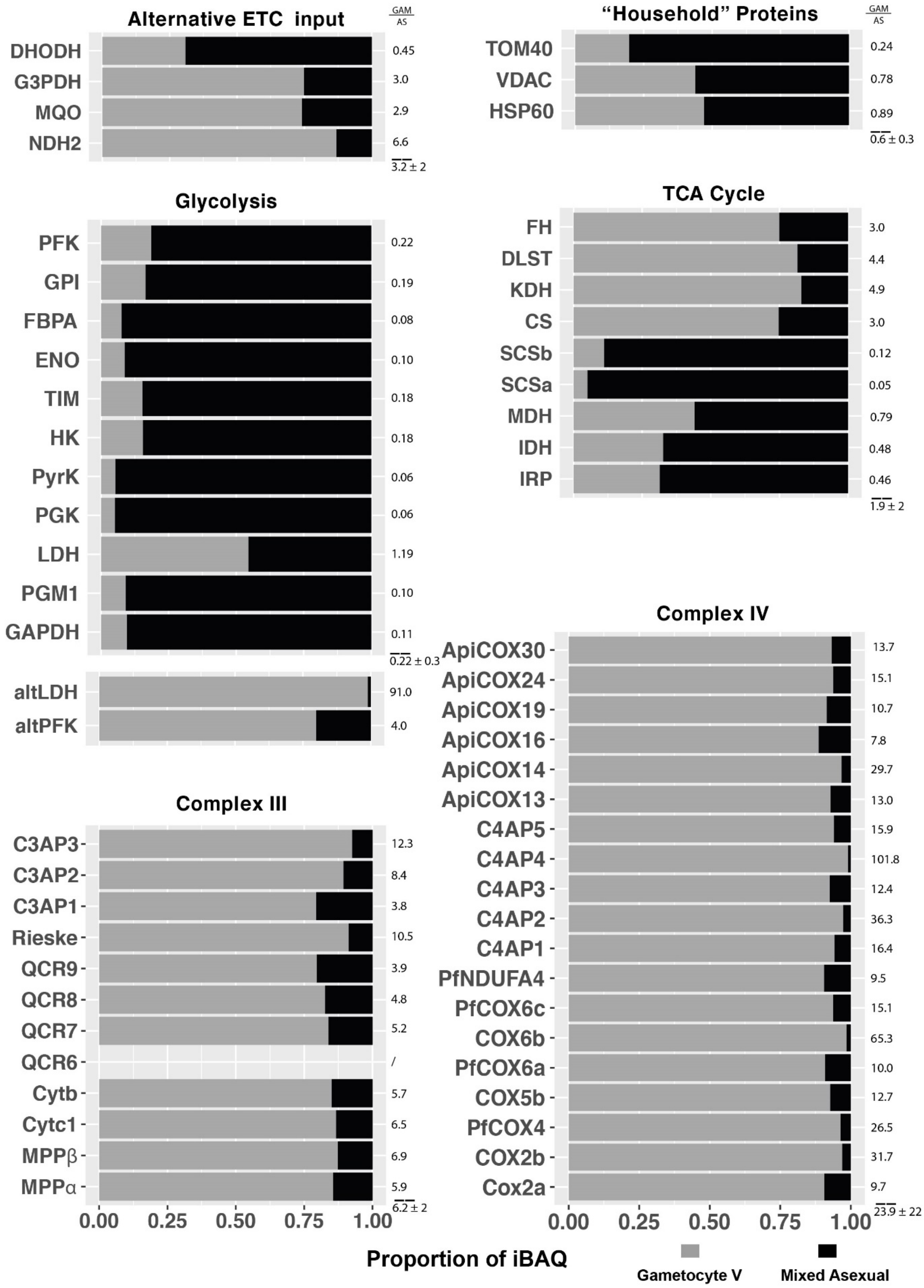

Supplementary Figure 10. Relative abundance comparisons of respiratory chain complexes III and IV and detected enzymes related to energy metabolism. Average total iBAQ values of four digitonin ABS parasite and gametocyte complexome samples, respectively, were calculated for individual proteins. VDAC, TOM40, and HSP60 were quantified to estimate degree of mitochondrial enrichment between stages.

Supplementary Table 1. Description of all samples analysed by complexome profiling.

Sample Name	Parasite Stage	Parasite strain	Detergent: Protein	Protein Quantity [µg]	Enrichment method
GCT1Da	Stage V Gametocyte	NF54	Digitonin 6:1	115	1
GCT1Db	Stage V Gametocyte	NF54/iGP2	Digitonin 6:1	100	1
GCT1Dc	Stage V Gametocyte	NF54/iGP2	Digitonin 6:1	180	1
GCT3D	Stage V Gametocyte	NF54/iGP2	Digitonin 4.5:1	400	3
ABS1Da	Mixed ABS	NF54/iGP2	Digitonin 6:1	150	1
ABS1Db	Mixed ABS	NF54/iGP2	Digitonin 6:1	150	1
ABS2D	Mixed ABS	NF54	Digitonin 6:1	150	2
ABS3D	Mixed ABS	NF54/iGP2	Digitonin 4.5:1	300	3
ABS1Ma	Mixed ABS	NF54/iGP2	DDM 3:1	150	1
ABS1Mb	Mixed ABS	NF54/iGP2	DDM 3:1	150	1
ABS2M	Mixed ABS	NF54	DDM 3:1	150	2
HMM200	Stage V Gametocyte	NF54/iGP2	Digitonin 6:1	200	3
HMM400	Stage V Gametocyte	NF54/iGP2	Digitonin 6:1	400	3

Supplementary Table 2. Abundance of putative supercomplex in asexual blood-stage parasites and gametocytes normalized separately. Intensities were normalized against highest value found in ABS3D (ABS) and GCT3D (GCT) respectively and averaged for all complex components. Relative abundance in putative supercomplex peaks compared to highest intensity value was expressed in %. A much higher proportion of intensity is found in supercomplex peaks in gametocytes compared to asexual blood stages.

		$CIII_2CIV$	$CIII_4$	$CIII_2CIV_2$	$CIII_4CIV$	$CIII_4CIV_2$
**ABS**	CIII	1.33%	2.54%	0.34%	0.06%	0.02%
	CIV	17.83%	4.50%	2.16%	1.00%	0.18%
**GCT**	CIII	2.77%	3.81%	1.00%	0.64%	0.26%
	CIV	26.67%	9.12%	12.25%	8.92%	4.24%

Supplementary Table 3 Conservation of transmembrane helices in complex III and complex IV. Table containing probability plots for transmembrane helices and inside and outside positioning of all AA residues of each putative complex III and complex IV protein in *P. falciparum* as well as their orthologues in *S. cerevisiae* and *H. sapiens* as generated by the TMHMM Server v. 2.0

**Complex III**	*Homo sapiens*	*Saccharomyces cerevisiae*	*Plasmodium falciparum*
MT-CYB	CYB_HUMAN 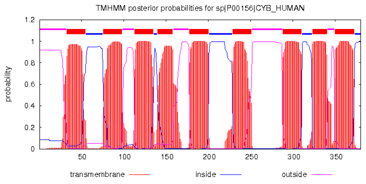	CYB_YEAST 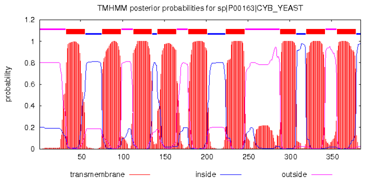	CYB_PLAFA 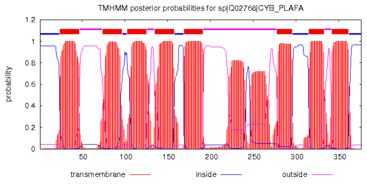
CYC1	CY1_HUMAN 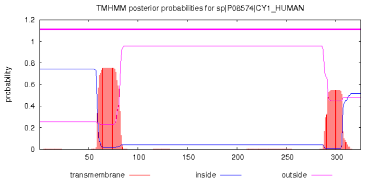	CY1_YEAST 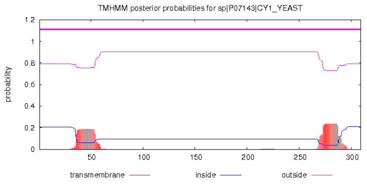	Q8I6U9_PLAF7 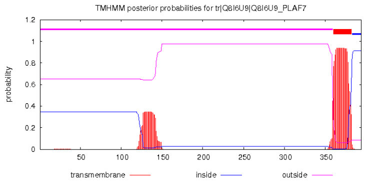
UQCRFS1	UCRI_HUMAN 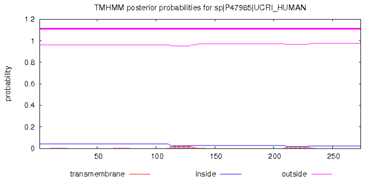	UCRI_YEAST 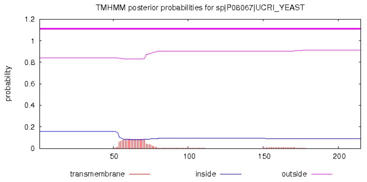	Q8IL75_PLAF7 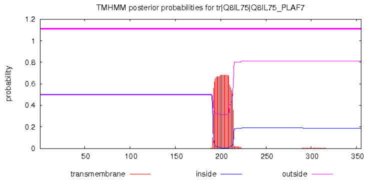
UQCRC1	QCR1_HUMAN 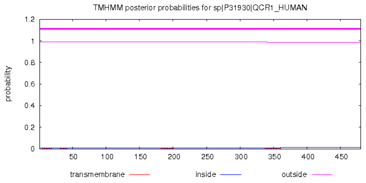	QCR1_YEAST 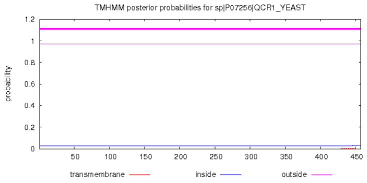	Q8I3N3_PLAF7 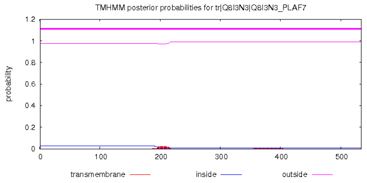
UQCRC2	QCR2_HUMAN 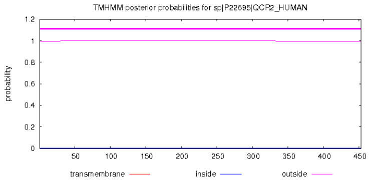	QCR2_YEAST 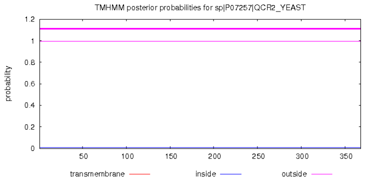	Q8I2I2_PLAF7 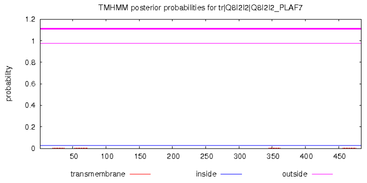

<table>
<tr><td>UQCRH</td><td>QCR6_HUMAN
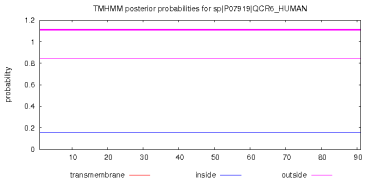
</td><td>QCR6_YEAST
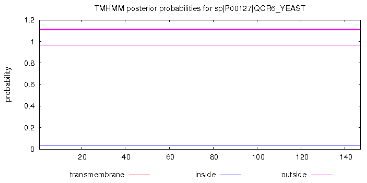
</td><td>Q8ILJ5_PLAF7
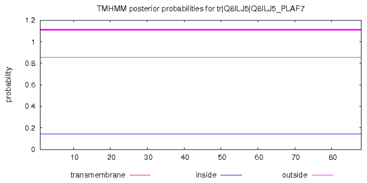
</td></tr>
<tr><td>UQCRB</td><td>QCR7_HUMAN
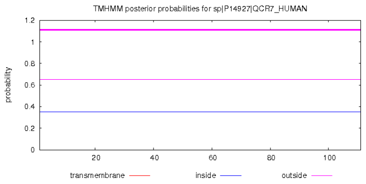
</td><td>QCR7_YEAST
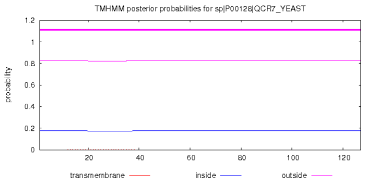
</td><td>Q8IJS2_PLAF7
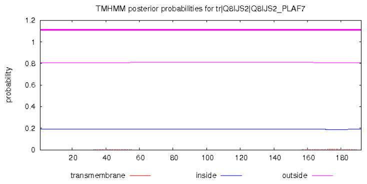
</td></tr>
<tr><td>UQCRQ</td><td>QCR8_HUMAN
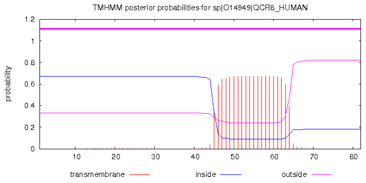
</td><td>QCR8_YEAST
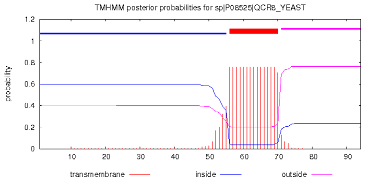
</td><td>Q9NLB3_PLAF7
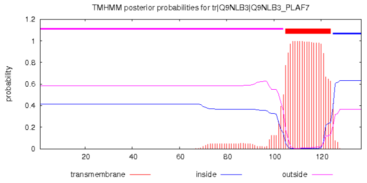
</td></tr>
<tr><td>UQCR10</td><td>QCR9_HUMAN
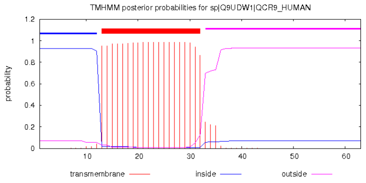
</td><td>QCR9_YEAST
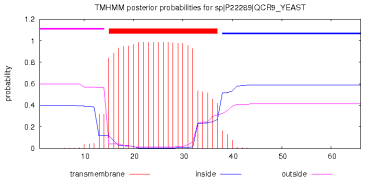
</td><td>C0H4H6_PLAF7
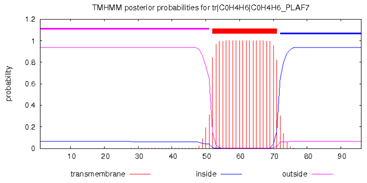
</td></tr>
<tr><td>UQCR11</td><td>QCR10_HUMAN
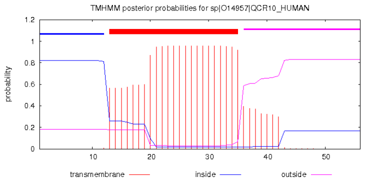
</td><td>QCR10_YEAST
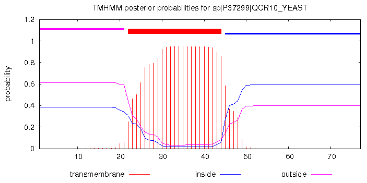
</td><td></td></tr>
<tr><td>PF3D7_0722700
C3AP1</td><td></td><td></td><td>Q8IBM6_PLAF7
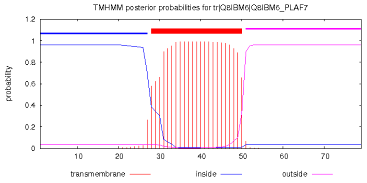
</td></tr>
</table>

PF3D7_1326000<br>C3AP2			A0A5K1K9E9_PLAF7<br>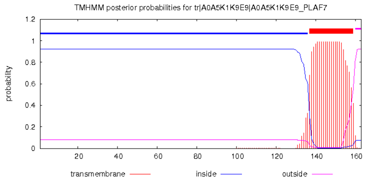
PF3D7_0817800<br>C3AP3			Q8IB13_PLAF7<br>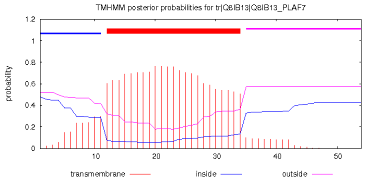

**Complex IV**	*Homo sapiens*	*Saccharomyces cerevisiae*	*Plasmodium falciparum*
MT-CO1	COX1_HUMAN 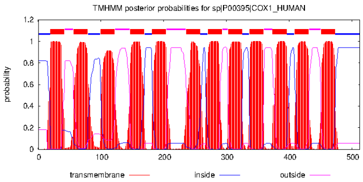	COX1_YEAST 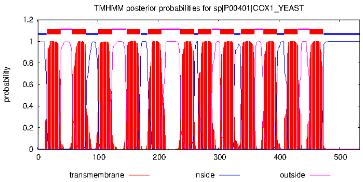	Q7HP04_PLAF7 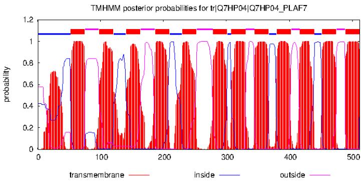
MT-CO2	COX2_HUMAN 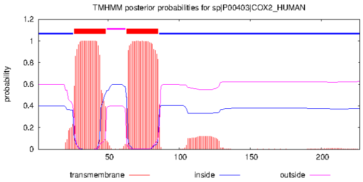	COX2_YEAST 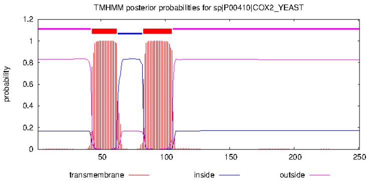	A0A5K1K9B3_PLAF7 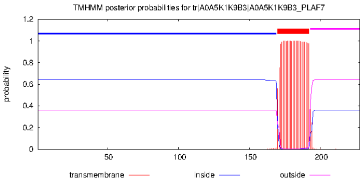 Q8I6V2_PLAF7 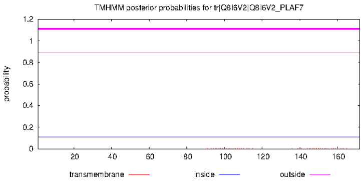
MT-CO3	COX3_HUMAN 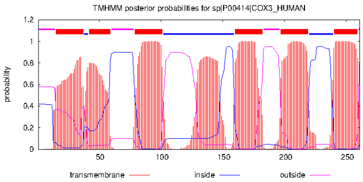	COX3_YEAST 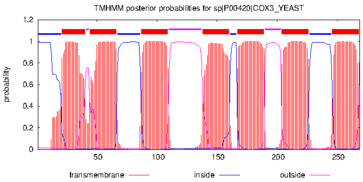	Q7HP05_PLAF7 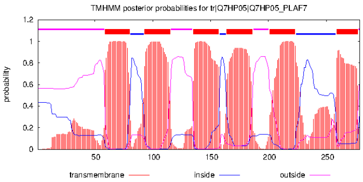
COX4I1	COX41_HUMAN 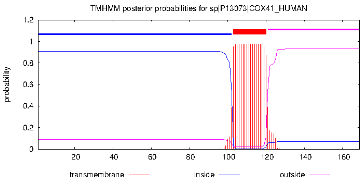	COX5B_YEAST 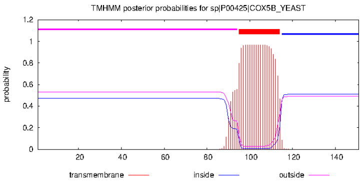	Q8IC02_PLAF7 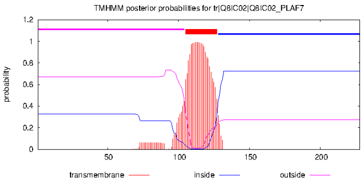
COX4I2	COX42_HUMAN 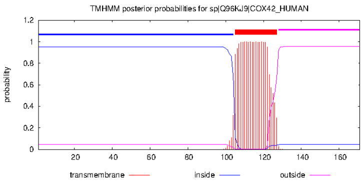	COX5A_YEAST 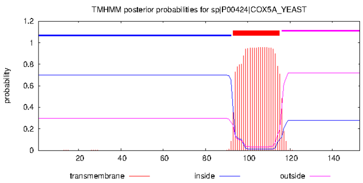	

<table>
<tr><td>COX5A</td><td>COX5A_HUMAN
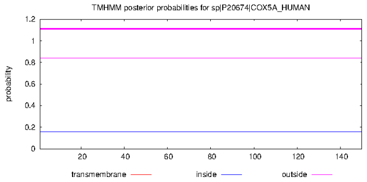</td><td>COX6_YEAST
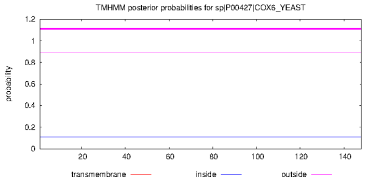</td><td></td></tr>
<tr><td>COX5B</td><td>COX5B_HUMAN
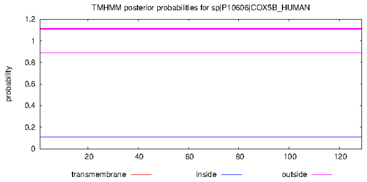</td><td>COX4_YEAST
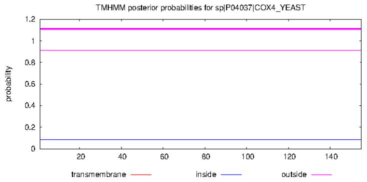</td><td>Q8I2N1_PLAF7
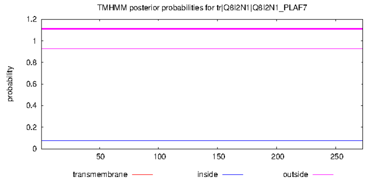</td></tr>
<tr><td>COX6A1</td><td>CX6A1_HUMAN
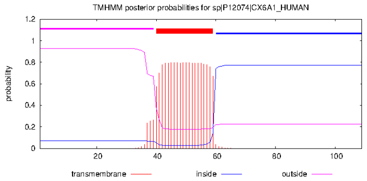</td><td rowspan="2">COX13_YEAST
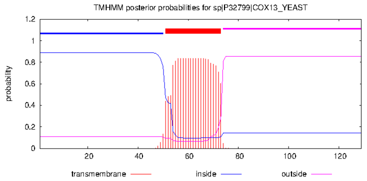</td><td rowspan="2">Q8IKI8_PLAF7
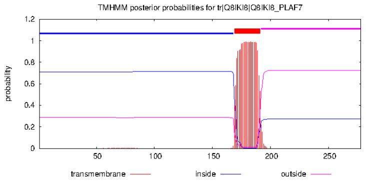</td></tr>
<tr><td>COX6A2</td><td>CX6A2_HUMAN
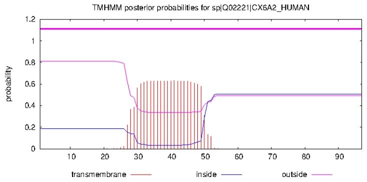</td></tr>
<tr><td>COX6B1</td><td>CX6B1_HUMAN
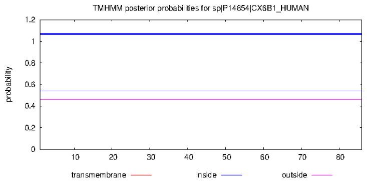</td><td rowspan="2">COX12_YEAST
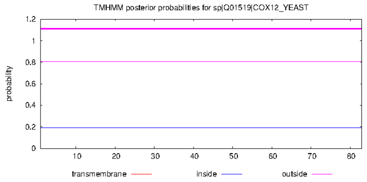</td><td rowspan="2">Q8I2M9_PLAF7
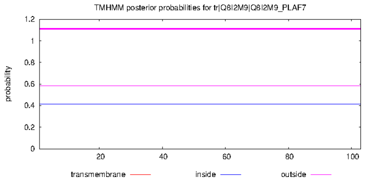</td></tr>
<tr><td>COX6B2</td><td>CX6B2_HUMAN
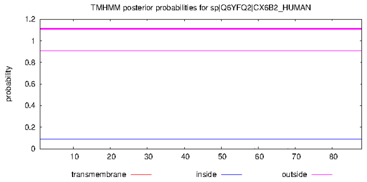</td></tr>
</table>

<table>
<tr><td>COX6C</td><td>COX6C_HUMAN
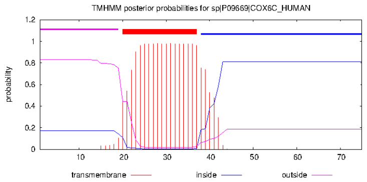</td><td>COX9_YEAST
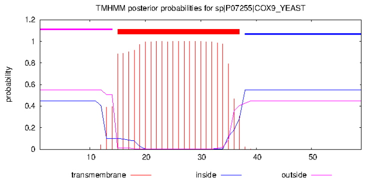</td><td>O97246_PLAF7
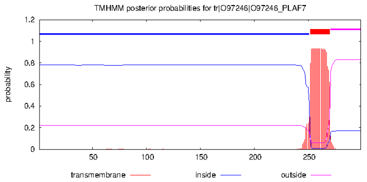</td></tr>
<tr><td>COX7A1</td><td>CX7A1_HUMAN
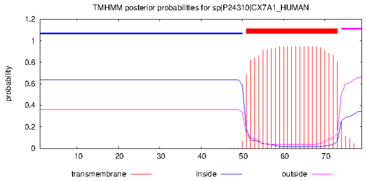</td><td rowspan="2">COX7_YEAST
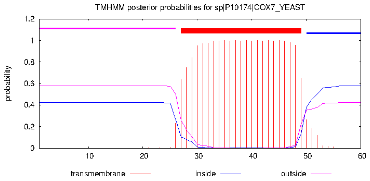</td><td rowspan="2"></td></tr>
<tr><td>COX7A2</td><td>CX7A2_HUMAN
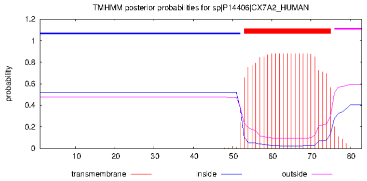</td></tr>
<tr><td>COX7B</td><td>COX7B_HUMAN
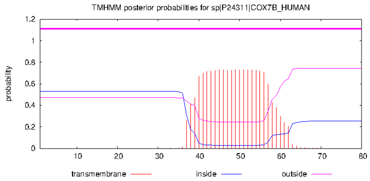</td><td></td><td></td></tr>
<tr><td>COX7B2</td><td>CX7B2_HUMAN
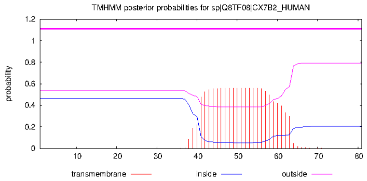</td><td></td><td></td></tr>
<tr><td>COX7C</td><td>COX7C_HUMAN
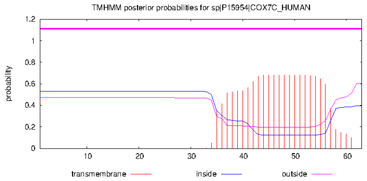</td><td>COX8_YEAST
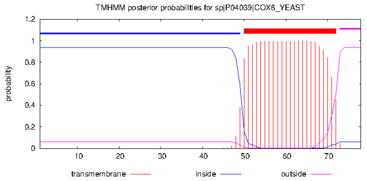</td><td></td></tr>
</table>

COX8A	COX8A_HUMAN 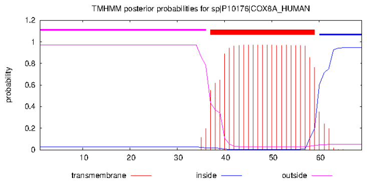		
COX8C	COX8C_HUMAN 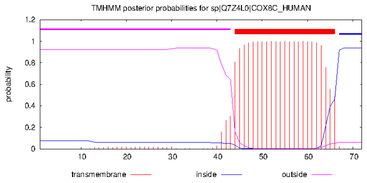		
NDUFA4	NDUA4_HUMAN 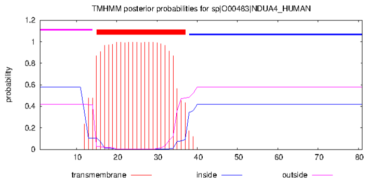	YP010_YEAST 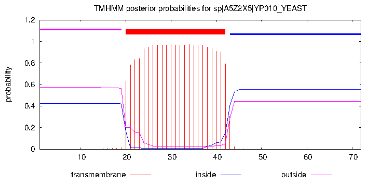	Q8IL73_PLAF7 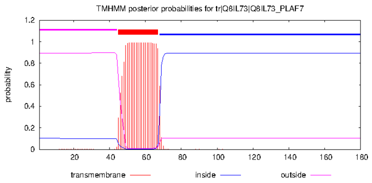
PF3D7_1022900 ApiCOX13	CISD3_HUMAN 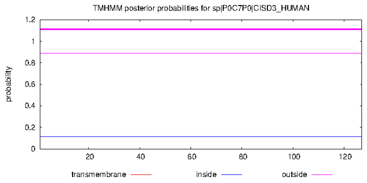		Q8IJH6_PLAF7 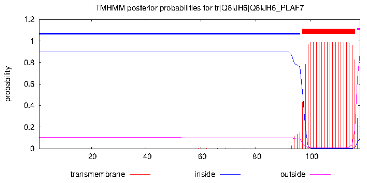
PF3D7_1339400 ApiCOX14			A0A5K1K8V5_PLAF7 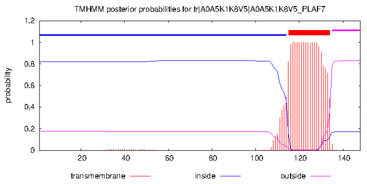
PF3D7_1345300 ApiCOX16			C0H5H3_PLAF7 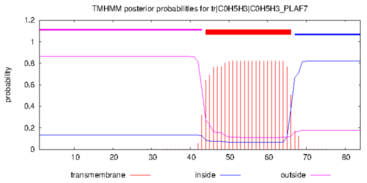

PF3D7_0523300<br>ApiCOX18			Q8I3N1_PLAF7<br>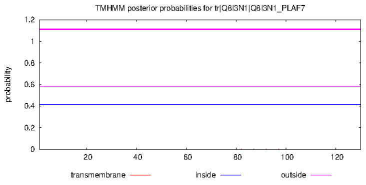
PF3D7_1402200<br>ApiCOX19			Q8IM67_PLAF7<br>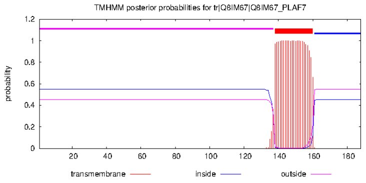
PF3D7_1362000<br>ApiCOX24			A0A5K1K922_PLAF7<br>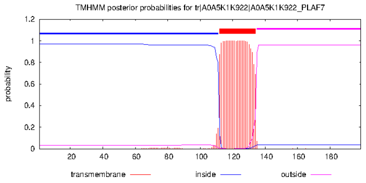
PF3D7_0915700<br>ApiCOX30			Q8I2Z5_PLAF7<br>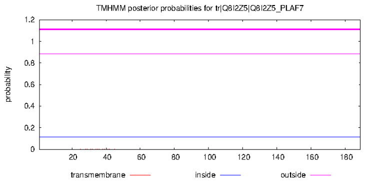
PF3D7_1125600<br>C4AP1			C6S3G9_PLAF7<br>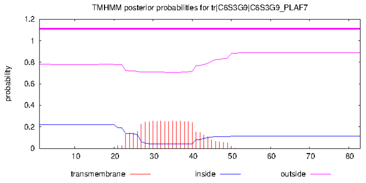
PF3D7_1025800<br>C4AP2			C6S3D5_PLAF7<br>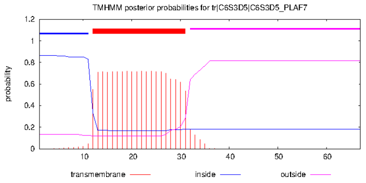

PF3D7_1003100<br>C4AP3			C6S3C0_PLAF7<br>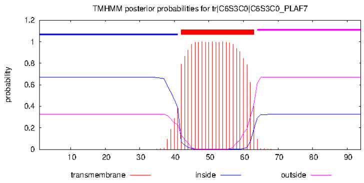
PF3D7_0608400<br>C4AP4			C6KST2_PLAF7<br>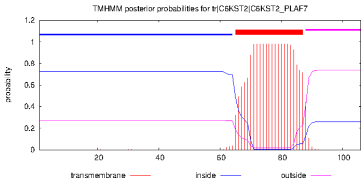
PF3D7_0809250<br>C4AP5			A0A5K1K8Z8_PLAF7<br>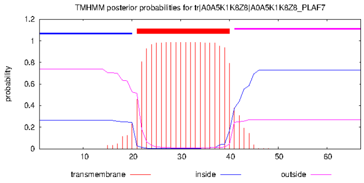

Supplementary References

1. Hartley AM, Meunier B, Pinotsis N, Marechal A. Rcf2 revealed in cryo-EM structures of hypoxic isoforms of mature mitochondrial III-IV supercomplexes. *Proc Natl Acad Sci USA* **117**, 9329-9337 (2020).
2. Krieger E, Vriend G. YASARA View - molecular graphics for all devices - from smartphones to workstations. *Bioinformatics* **20,** 2981-2982 (2014).
3. Wittig I, Beckhaus T, Wumaier Z, Karas M, Schägger H. Mass estimation of native proteins by blue native electrophoresis: principles and practical hints. *Mol Cell Proteomics* **9**, 2149-2161 (2010).
