# Supplementary material for: Composition and stage dynamics of mitochondrial complexes in Plasmodium falciparum
Source: Nat Commun. 2021 Jun 21;12:3820. doi: 10.1038/s41467-021-23919-x (PMC8217502; doi:10.1038/s41467-021-23919-x)
Supplement: Supplementary file 3 — Description of Additional Supplementary Files [file 41467_2021_23919_MOESM3_ESM.docx]

Description of Additional Supplementary Files

Title: Supplementary Information

PDF containing Supplementary Figures 1-10, Supplementary Tables 1-3 and accompanying Supplementary References.

Title: Supplementary Data 1

Sheet1 “Processed MS Data”: MS data of all proteins identified in all complexome profiles in this study
Sheet2 ”Analyzed Complexes”: MS data of all proteins discussed in this study in all complexome profiles
Sheet3 “Mass calibration” : Information on mass calibration for all complexome profiles

Title: Supplementary Data 2

Sheet1 “HMM OXPHOS Data”: MS data all proteins identified in high molecular mass profiles assigned to CIII, CIV and CV
Sheet2 “HMM Mass Calibration”: Information on mass calibration for all high molecular mass profiles

Title: Supplementary Data 3

Description: MS data of all proteins identified whole cell lysate SDS profiles of asexual blood stages and gametocytes

Title: Source Data

Description: Excel file containing six sheets with underlying data for figures 2-7. For sheets containing source data for complexome profiling data (figure 2, 3 and 5), underlying iBAQ values and normalized values for all proteins that are shown in the respective figure, are given. This includes data for samples that were not shown in the respective figure. If averages for a complex were shown in the figure, numerical values for each datapoint are supplied in the sheet. For figure 4 a matrix containing gene IDs for all genes underlying the circles in the figure is supplied. For abundance comparisons (figure 6 and figure 7), underlying iBAQ value sums are given as well as all abundances in each gel slice. While for the figure only iBAQ values at sizes that match predicted molecular mass of proteins, the sheet also provides the fold changes and iBAQ sums you would get if you summed abundances in all slices.
