# Supplementary material for: Composition and stage dynamics of mitochondrial complexes in Plasmodium falciparum
Source: Nat Commun. 2021 Jun 21;12:3820. doi: 10.1038/s41467-021-23919-x (PMC8217502; doi:10.1038/s41467-021-23919-x)
Supplement: Supplementary file 7 — Reporting Summary [file 41467_2021_23919_MOESM7_ESM.pdf]

nature research

Corresponding author(s): Taco W.A. Kooij

Last updated by author(s): May 5, 2021

Reporting Summary

Nature Research wishes to improve the reproducibility of the work that we publish. This form provides structure for consistency and transparency in reporting. For further information on Nature Research policies, see our Editorial Policies and the Editorial Policy Checklist.

Statistics

For all statistical analyses, confirm that the following items are present in the figure legend, table legend, main text, or Methods section.

n/a	Confirmed	
x		The exact sample size (n) for each experimental group/condition, given as a discrete number and unit of measurement
x		A statement on whether measurements were taken from distinct samples or whether the same sample was measured repeatedly
x		The statistical test(s) used AND whether they are one- or two-sided<br>*Only common tests should be described solely by name; describe more complex techniques in the Methods section.*
x		A description of all covariates tested
x		A description of any assumptions or corrections, such as tests of normality and adjustment for multiple comparisons
	x	A full description of the statistical parameters including central tendency (e.g. means) or other basic estimates (e.g. regression coefficient) AND variation (e.g. standard deviation) or associated estimates of uncertainty (e.g. confidence intervals)
x		For null hypothesis testing, the test statistic (e.g. F, t, r) with confidence intervals, effect sizes, degrees of freedom and P value noted<br>*Give P values as exact values whenever suitable.*
x		For Bayesian analysis, information on the choice of priors and Markov chain Monte Carlo settings
x		For hierarchical and complex designs, identification of the appropriate level for tests and full reporting of outcomes
	x	Estimates of effect sizes (e.g. Cohen's d, Pearson's r), indicating how they were calculated

Our web collection on statistics for biologists contains articles on many of the points above.

Software and code

Policy information about availability of computer code

Data collection	Raw MS data files from all samples were collected using Xcalibur (Version 3.1)
Data analysis	Raw MS data files from all samples were analysed using MaxQuant (v1.5.0.25) and searched against a P. falciparum reference proteome (isolate 3D7, version March 21, 2020). Protein migration profiles were clustered using Cluster3.0. MS data was processed in Microsoft Excel. MS data was further processed R and visualized using the package ggplot2. HHPred (Webserver based on HH-suite 3.0), BLAST, jackhmmer (HmmerWeb version 2.41.1) were used for protein homology detection.

For manuscripts utilizing custom algorithms or software that are central to the research but not yet described in published literature, software must be made available to editors and reviewers. We strongly encourage code deposition in a community repository (e.g. GitHub). See the Nature Research guidelines for submitting code & software for further information.

Data

Policy information about availability of data

All manuscripts must include a data availability statement. This statement should provide the following information, where applicable:

- Accession codes, unique identifiers, or web links for publicly available datasets
- A list of figures that have associated raw data
- A description of any restrictions on data availability

All raw and processed complexome data generated in this study was deposited at the ComplexomE profiling DAta Resource (CEDAR) and can be retrieved under www3.cmbi.umcn.nl/cedar/browse/experiments/CRX23. Source data are provided with this paper. Complete underlying datasets for figures 3,5,6 and 7 and further contextually relevant data are provided in Supplementary Data 1.

Field-specific reporting

Please select the one below that is the best fit for your research. If you are not sure, read the appropriate sections before making your selection.

- [x] Life sciences
- [ ] Behavioural & social sciences
- [ ] Ecological, evolutionary & environmental sciences

For a reference copy of the document with all sections, see nature.com/documents/nr-reporting-summary-flat.pdf

Life sciences study design

All studies must disclose on these points even when the disclosure is negative.

Sample size	Not applicable because a single complexome experiment always involves pooling of parasite material from multiple cultures until sufficient protein quantity is reached for an experiment.
Data exclusions	A peptide for protein group Cox2a underlying quantification in Figure 6 was removed due to inconsistent migration pattern, making it likely to be a false positive. This is disclosed in lines 428-430. As a pilot to test viability of the complexome profiling, the experiment was performed on insufficent amounts of material and a different fraction. This data was not included as it did not contain any relevant information. Data from pilot experiment is available upon request.
Replication	For both life cycle stages multiple replicates were performed under different conditions. In total seven asexual blood stage samples and six gametocyte samples were analyzed. GCT1Db/GCT1Dc, ABS1Da/ABS1Db and ABS1Ma/ABS1Mb represent technical replicate pairings while the remainder are distinct samples. Consistency and/or differences between samples were monitored and where relevant discussed. All collected data can be viewed in Supplementary Information S1. No replicates were omitted from the submission. A typical power calculation is not applicable
Randomization	Samples were not randomized during data collection or analysis. We are not aware of any biases this might have introduced and it would not have been feasible in this context. Due to inherent big differences between life cycle stages randomization would have been immediately obvious to the investigator.
Blinding	Investigators were not blinded during data collection or analysis. We are not aware of any biases this might have introduced and it would not have been feasible in this context. Due to inherent big differences between life cycle stages blinding would have been immediately obvious to the investigator.

Reporting for specific materials, systems and methods

We require information from authors about some types of materials, experimental systems and methods used in many studies. Here, indicate whether each material, system or method listed is relevant to your study. If you are not sure if a list item applies to your research, read the appropriate section before selecting a response.

Materials & experimental systems

n/a	Involved in the study
x	Antibodies
	x Eukaryotic cell lines
x	Palaeontology and archaeology
x	Animals and other organisms
x	Human research participants
x	Clinical data
x	Dual use research of concern

Methods

n/a	Involved in the study
x	ChIP-seq
x	Flow cytometry
x	MRI-based neuroimaging

Eukaryotic cell lines

Policy information about cell lines

Cell line source(s)	NF54; NF54-igp2
Authentication	NF54: Originally published in this institute and an authenticated master cell bank is used from which we regularly start new cultures; NF54-igp2 was directly provided by our coauthors Till Voss and Sylwia Boltryk that developed the line recently.
Mycoplasma contamination	Parasites are routinely controlled for mycoplasma contamination by PCR.
Commonly misidentified lines (See ICLAC register)	n/a
